# Supplementary material for: Tissue and regional expression patterns of dicistronic tRNA–mRNA transcripts in grapevine (Vitis vinifera) and their evolutionary co-appearance with vasculature in land plants
Source: Hortic Res. 2021 Jun 1;8:137. doi: 10.1038/s41438-021-00572-5 (PMC8166872; doi:10.1038/s41438-021-00572-5)
Supplement: Supplementary file 1 — Supplemental File 1 [file 41438_2021_572_MOESM1_ESM.docx]

Alignment of sequencing results from PCR product for two dicistronic tRNA-mRNA candidates to the expected PCR product.

Query: tRNA-Pro-TGG-2-9_Intergenic_region Query ID: lcl|Query_54328 Length: 171

>1_ProC_F_B09

Sequence ID: Query_54330 Length: 125

Range 1: 1 to 124

Score:224 bits(121), Expect:9e-64,

Identities:123/124(99%), Gaps:0/124(0%), Strand: Plus/Plus

Query 48 TTTCTTTCGCTGGGTTTTGGTTTTACTTCACCATAAACCTCAAAAAAGCCCTCTTATGCT 107

||||||| ||||||||||||||||||||||||||||||||||||||||||||||||||||

Sbjct 1 TTTCTTTGGCTGGGTTTTGGTTTTACTTCACCATAAACCTCAAAAAAGCCCTCTTATGCT 60

Query 108 CTTCTGCAAATTTCATTTGTGTTATTGGTACTGAAACTCCGAGGCGGTGGCAGGCAAGGA 167

||||||||||||||||||||||||||||||||||||||||||||||||||||||||||||

Sbjct 61 CTTCTGCAAATTTCATTTGTGTTATTGGTACTGAAACTCCGAGGCGGTGGCAGGCAAGGA 120

Query 168 AACA 171

||||

Sbjct 121 AACA 124

Query: tRNA-Pro-TGG-2-9_Intergenic_region Query ID: lcl|Query_54328 Length: 171

>2_ProC_R_B10

Sequence ID: Query_54331 Length: 120

Range 1: 3 to 120

Score:213 bits(115), Expect:2e-60,

Identities:118/119(99%), Gaps:1/119(0%), Strand: Plus/Minus

Query 1 TGCGAGAGGTCCCGAGTTCGATTCTCGGAATGCCCCAATCTTTTTATTTTCTTTCGCTGG 60

||||||||||||||||||||||||||||||||||||||||||||||||||||||||||||

Sbjct 120 TGCGAGAGGTCCCGAGTTCGATTCTCGGAATGCCCCAATCTTTTTATTTTCTTTCGCTGG 61

Query 61 GTTTTGGTTTTACTTCACCATAAACCTCAAAAAAGCCCTCTTATGCTCTTCTGCAAATT 119

||||||||||||||||||||||||||||||||||||||||||||||||| |||||||||

Sbjct 60 GTTTTGGTTTTACTTCACCATAAACCTCAAAAAAGCCCTCTTATGCTCT-CTGCAAATT 3

Query: Val_intergenic_PCR_Product Query ID: lcl|Query_16750 Length: 376

>5_ValC_F_C01

Sequence ID: Query_16752 Length: 320

Range 1: 1 to 320

Score:586 bits(317), Expect:6e-172,

Identities:319/320(99%), Gaps:0/320(0%), Strand: Plus/Plus

Query 56 ATTGCCAGAGTCTTCCATTTCTGTTGGGAGTCTCCCAGGGTCAGAGTATCAACGACACTC 115

||||||| ||||||||||||||||||||||||||||||||||||||||||||||||||||

Sbjct 1 ATTGCCACAGTCTTCCATTTCTGTTGGGAGTCTCCCAGGGTCAGAGTATCAACGACACTC 60

Query 116 AGTGCCACAATCTCATTTCCATTTCTGCTAGGAGTCTCCCAGATTCTCAATATCAACAAC 175

||||||||||||||||||||||||||||||||||||||||||||||||||||||||||||

Sbjct 61 AGTGCCACAATCTCATTTCCATTTCTGCTAGGAGTCTCCCAGATTCTCAATATCAACAAC 120

Query 176 ACTCACCCAGATTTTTAACATTTTCTCATCTGGATGTTCATCAATTAGTCAAACAATGCA 235

||||||||||||||||||||||||||||||||||||||||||||||||||||||||||||

Sbjct 121 ACTCACCCAGATTTTTAACATTTTCTCATCTGGATGTTCATCAATTAGTCAAACAATGCA 180

Query 236 GATTCAGCCACACCCACTTCCAAACTATAGTCTTAGGTCACCAATTTTCTCACCTTCGAC 295

||||||||||||||||||||||||||||||||||||||||||||||||||||||||||||

Sbjct 181 GATTCAGCCACACCCACTTCCAAACTATAGTCTTAGGTCACCAATTTTCTCACCTTCGAC 240

Query 296 CCATTTTCTCATACCTTTCTCATCATCATTCCAACCTACTAAACTTGTCTCTATTCCAAA 355

||||||||||||||||||||||||||||||||||||||||||||||||||||||||||||

Sbjct 241 CCATTTTCTCATACCTTTCTCATCATCATTCCAACCTACTAAACTTGTCTCTATTCCAAA 300

Query 356 TTTCCCATCTGGGTCTTGCG 375

||||||||||||||||||||

Sbjct 301 TTTCCCATCTGGGTCTTGCG 320

Query: Val_intergenic_PCR_Product Query ID: lcl|Query_16750 Length: 376

>6_ValC_R_C02

Sequence ID: Query_16753 Length: 307

Range 1: 1 to 307

Score:562 bits(304), Expect:1e-164,

Identities:306/307(99%), Gaps:0/307(0%), Strand: Plus/Minus

Query 1 CACTAGAGGTCCCCGGTTCGAACCCGGGCTCAGACATTTGCATTTTTATTTTATTATTGC 60

||||||||||||||||||||||||||||||||||||||||||||||||||||||||||||

Sbjct 307 CACTAGAGGTCCCCGGTTCGAACCCGGGCTCAGACATTTGCATTTTTATTTTATTATTGC 248

Query 61 CAGAGTCTTCCATTTCTGTTGGGAGTCTCCCAGGGTCAGAGTATCAACGACACTCAGTGC 120

|| |||||||||||||||||||||||||||||||||||||||||||||||||||||||||

Sbjct 247 CACAGTCTTCCATTTCTGTTGGGAGTCTCCCAGGGTCAGAGTATCAACGACACTCAGTGC 188

Query 121 CACAATCTCATTTCCATTTCTGCTAGGAGTCTCCCAGATTCTCAATATCAACAACACTCA 180

||||||||||||||||||||||||||||||||||||||||||||||||||||||||||||

Sbjct 187 CACAATCTCATTTCCATTTCTGCTAGGAGTCTCCCAGATTCTCAATATCAACAACACTCA 128

Query 181 CCCAGATTTTTAACATTTTCTCATCTGGATGTTCATCAATTAGTCAAACAATGCAGATTC 240

||||||||||||||||||||||||||||||||||||||||||||||||||||||||||||

Sbjct 127 CCCAGATTTTTAACATTTTCTCATCTGGATGTTCATCAATTAGTCAAACAATGCAGATTC 68

Query 241 AGCCACACCCACTTCCAAACTATAGTCTTAGGTCACCAATTTTCTCACCTTCGACCCATT 300

||||||||||||||||||||||||||||||||||||||||||||||||||||||||||||

Sbjct 67 AGCCACACCCACTTCCAAACTATAGTCTTAGGTCACCAATTTTCTCACCTTCGACCCATT 8

Query 301 TTCTCAT 307

|||||||

Sbjct 7 TTCTCAT 1
