# Supplementary material for: Tissue and regional expression patterns of dicistronic tRNA–mRNA transcripts in grapevine (Vitis vinifera) and their evolutionary co-appearance with vasculature in land plants
Source: Hortic Res. 2021 Jun 1;8:137. doi: 10.1038/s41438-021-00572-5 (PMC8166872; doi:10.1038/s41438-021-00572-5)
Supplement: Supplementary file 2 — Supplemental Fig S1 [file 41438_2021_572_MOESM2_ESM.pdf]

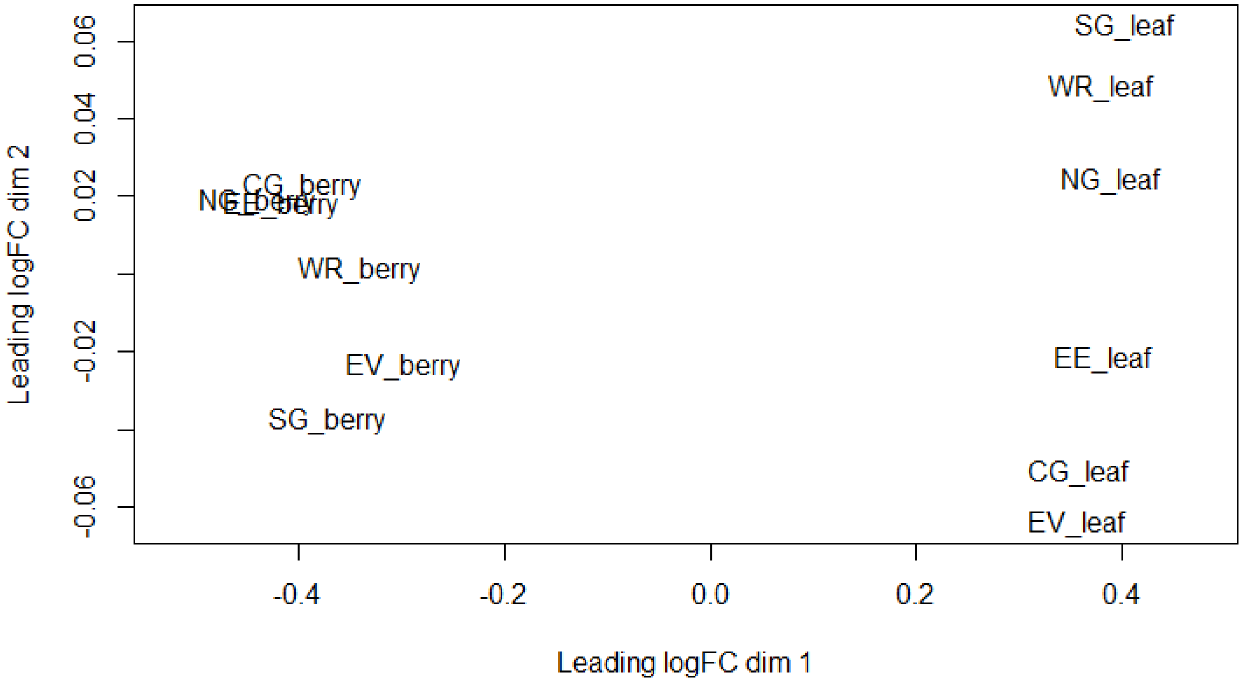

Supplemental_Fig_S1.pdf: MDS plots of total raw counts of all tRNAs expressed in leaf and berry samples for each sub-region.
