# Supplementary material for: Tissue and regional expression patterns of dicistronic tRNA–mRNA transcripts in grapevine (Vitis vinifera) and their evolutionary co-appearance with vasculature in land plants
Source: Hortic Res. 2021 Jun 1;8:137. doi: 10.1038/s41438-021-00572-5 (PMC8166872; doi:10.1038/s41438-021-00572-5)
Supplement: Supplementary file 3 — Supplemental Fig S2 [file 41438_2021_572_MOESM3_ESM.pdf]

a)

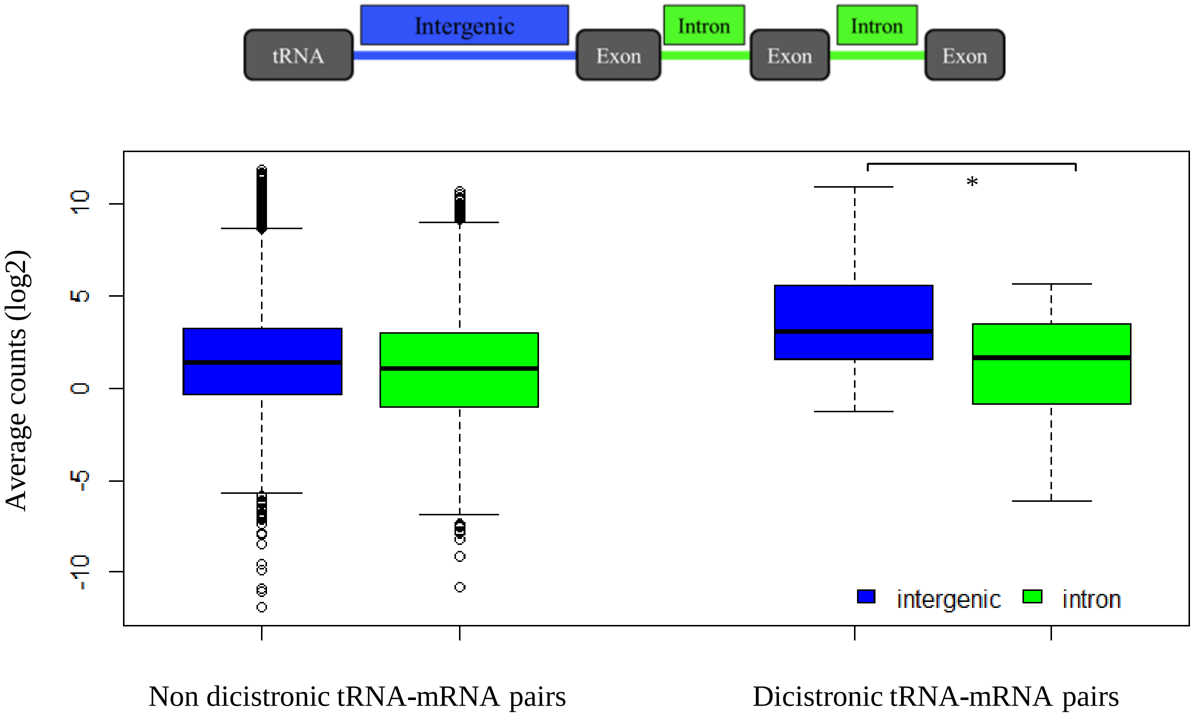

b)

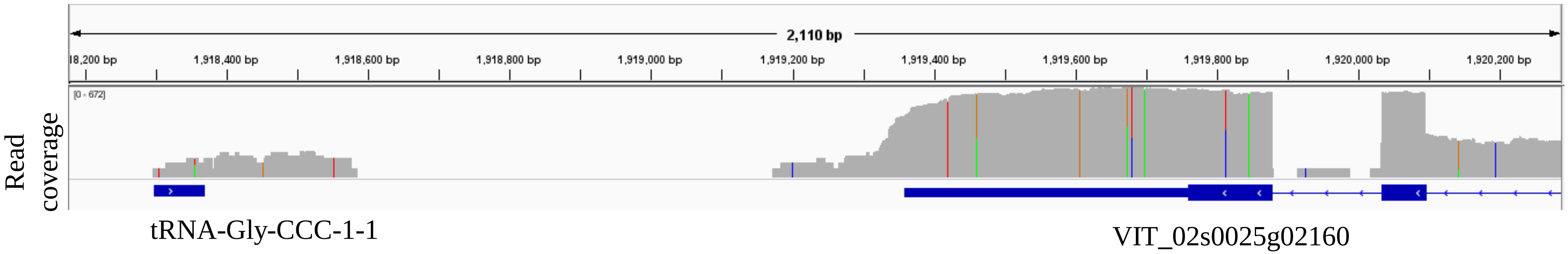

Supplemental_Fig_S2.pdf: Comparative analysis of expression levels of tRNA-mRNA intergenic regions (blue) and closest two introns (green) in non-dicistronic (left) and putative dicistronic tRNA-mRNA pairs (right) in leaf tissue. a) Average expression of intergenic region was significantly higher than the two closest introns in dicistronic tRNA-mRNA pairs (t-test, p-value < 0.05). b) Genome browser view of a non-dicistronic tRNA-mRNA pair (tRNA-Gly-CCC-1-1_VIT_02s0025g02160) identified by the DiRT pipeline. In this example, the tRNA and PCG are both expressed but the intergenic region does not present continuous coverage.
