# Supplementary material for: Tissue and regional expression patterns of dicistronic tRNA–mRNA transcripts in grapevine (Vitis vinifera) and their evolutionary co-appearance with vasculature in land plants
Source: Hortic Res. 2021 Jun 1;8:137. doi: 10.1038/s41438-021-00572-5 (PMC8166872; doi:10.1038/s41438-021-00572-5)
Supplement: Supplementary file 4 — Supplemental Fig S3 [file 41438_2021_572_MOESM4_ESM.pdf]

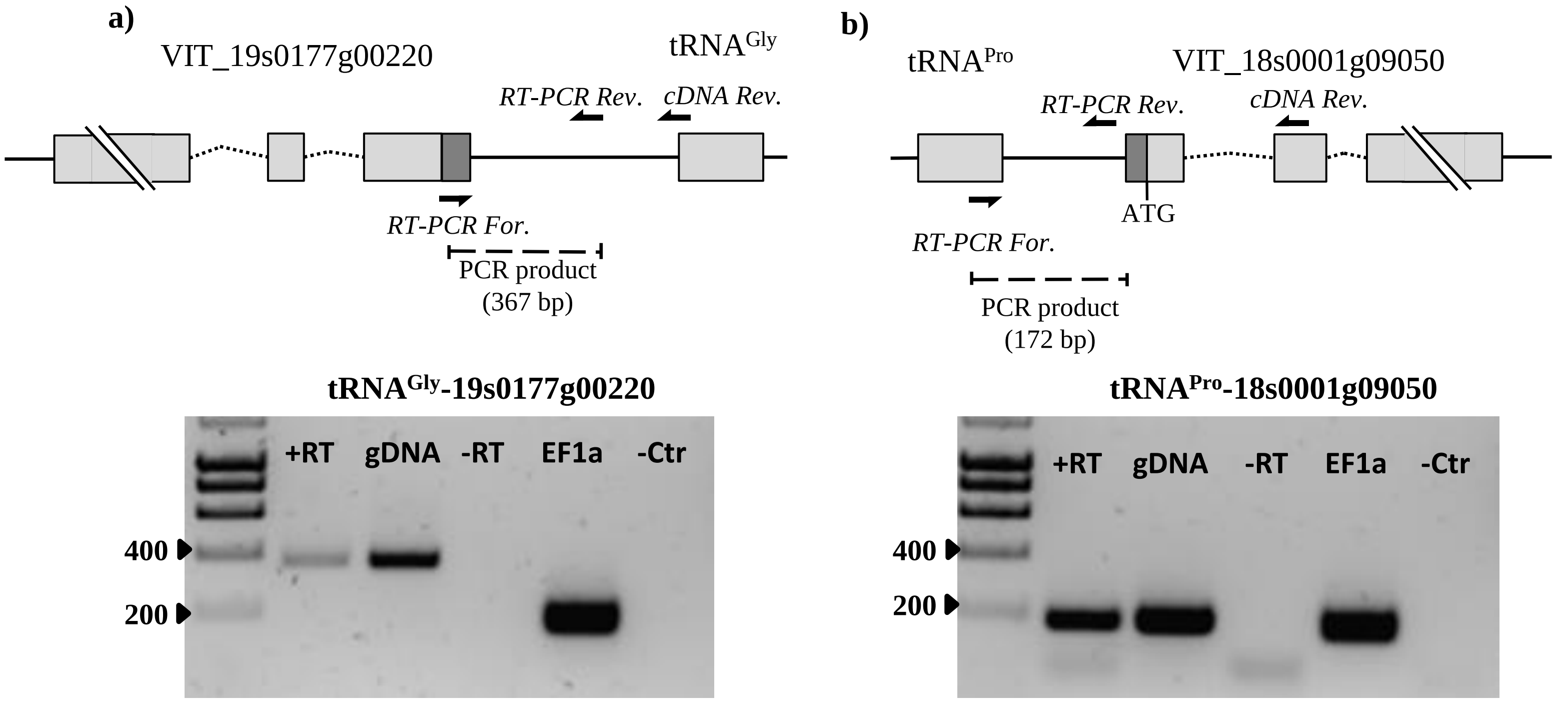

Supplemental_Fig_S3.pdf: RT-PCR confirmation of identified dicistronic transcripts in berry samples. Model of putative dicistronic tRNA-mRNA transcript showing primers used for cDNA synthesis (cDNA rev.) and for the PCR reaction (RT-PCR For and RT-PCR Rev). Confirmation of actively transcribed intergenic region through RT-PCR for candidates a) $tRNA^{GlyCCC}$_VIT_19s0177g00220 (367 bp) and b) $tRNA^{ProTGG}$-VIT_18s0001g09050 (172 bp). +RT: cDNA as template, gDNA: genomic DNA was used as a control, -RT: RT-PCR negative control, EF1a: Elongation Factor 1-alpha was used as a positive control (150 bp), -Ctr: PCR negative control.
