# Supplementary material for: Tissue and regional expression patterns of dicistronic tRNA–mRNA transcripts in grapevine (Vitis vinifera) and their evolutionary co-appearance with vasculature in land plants
Source: Hortic Res. 2021 Jun 1;8:137. doi: 10.1038/s41438-021-00572-5 (PMC8166872; doi:10.1038/s41438-021-00572-5)
Supplement: Supplementary file 5 — Supplemental Fig S4 [file 41438_2021_572_MOESM5_ESM.pdf]

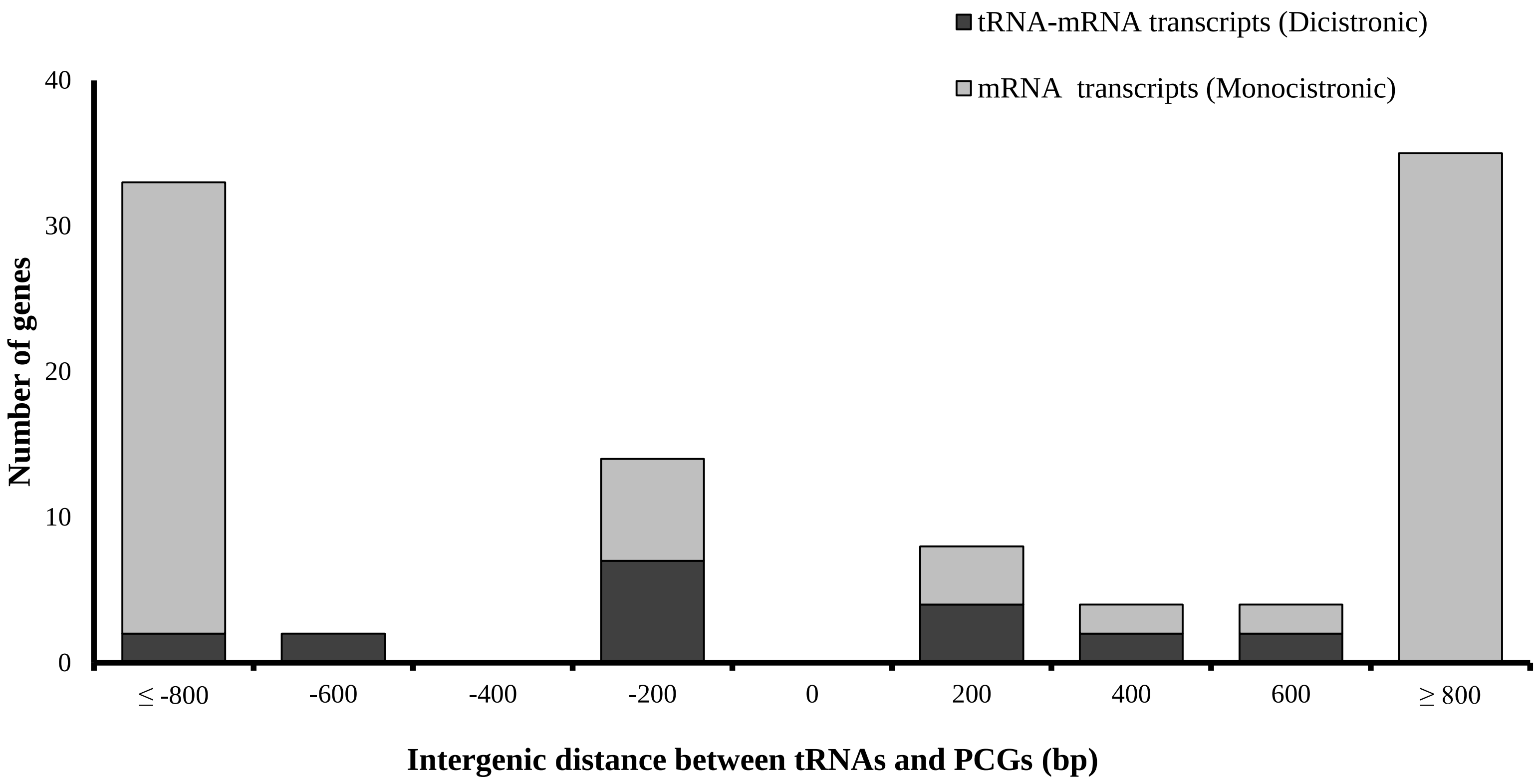

Supplemental_Fig_S4.pdf: Frequency of the length of the intergenic distance between tRNA (centred at zero) and protein coding genes (PGCs) for dicistronic and monocistronic transcripts. Vertical bars show the number of neighbouring tRNAs and PCGs pairs forming putative dicistronic tRNA-mRNA transcripts (black), or monocistronic transcripts (grey).
