# Supplementary material for: Tissue and regional expression patterns of dicistronic tRNA–mRNA transcripts in grapevine (Vitis vinifera) and their evolutionary co-appearance with vasculature in land plants
Source: Hortic Res. 2021 Jun 1;8:137. doi: 10.1038/s41438-021-00572-5 (PMC8166872; doi:10.1038/s41438-021-00572-5)
Supplement: Supplementary file 6 — Supplemental Fig S5 [file 41438_2021_572_MOESM6_ESM.pdf]

a)

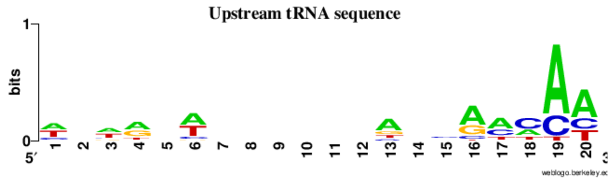

b)

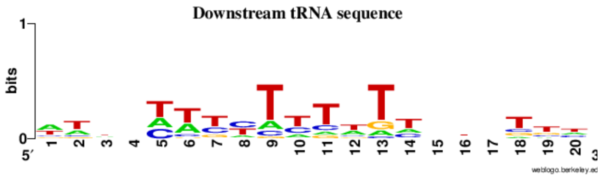

Supplemental_Fig_S5.pdf: Weblogo sequence analysis of the first 20 bp a) upstream; b) downstream of the 19 candidates dicistronic tRNAs.
