# Supplementary material for: Tissue and regional expression patterns of dicistronic tRNA–mRNA transcripts in grapevine (Vitis vinifera) and their evolutionary co-appearance with vasculature in land plants
Source: Hortic Res. 2021 Jun 1;8:137. doi: 10.1038/s41438-021-00572-5 (PMC8166872; doi:10.1038/s41438-021-00572-5)
Supplement: Supplementary file 9 — Supplemental Fig S8 [file 41438_2021_572_MOESM9_ESM.pdf]

a)

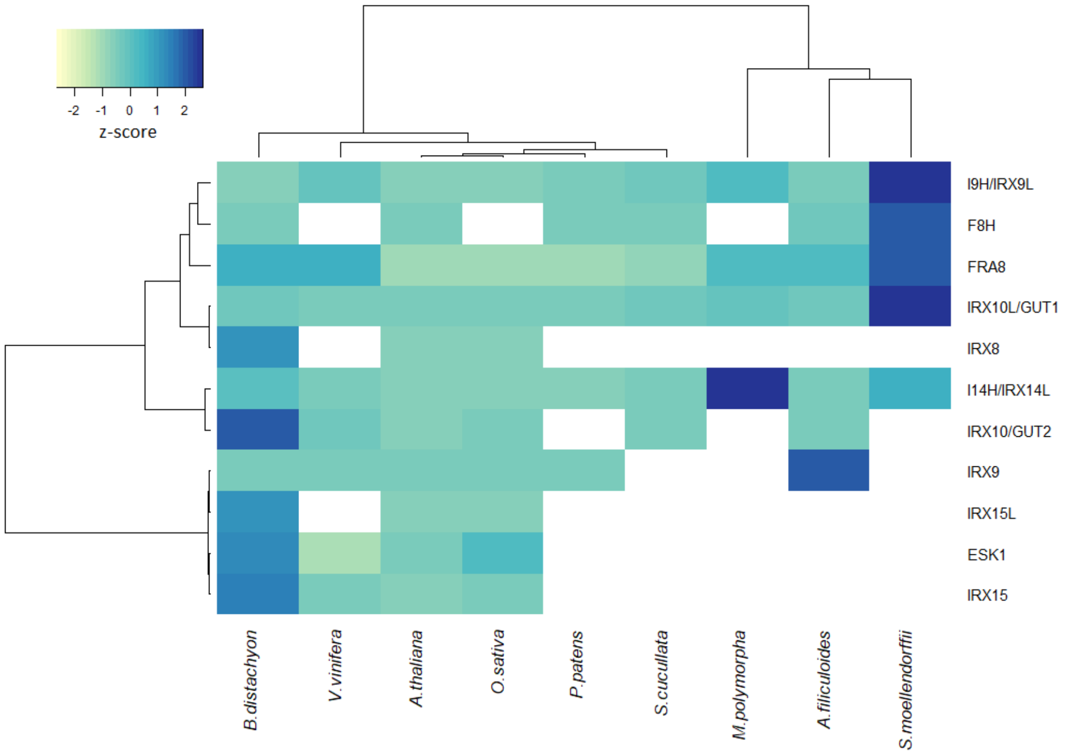

b)

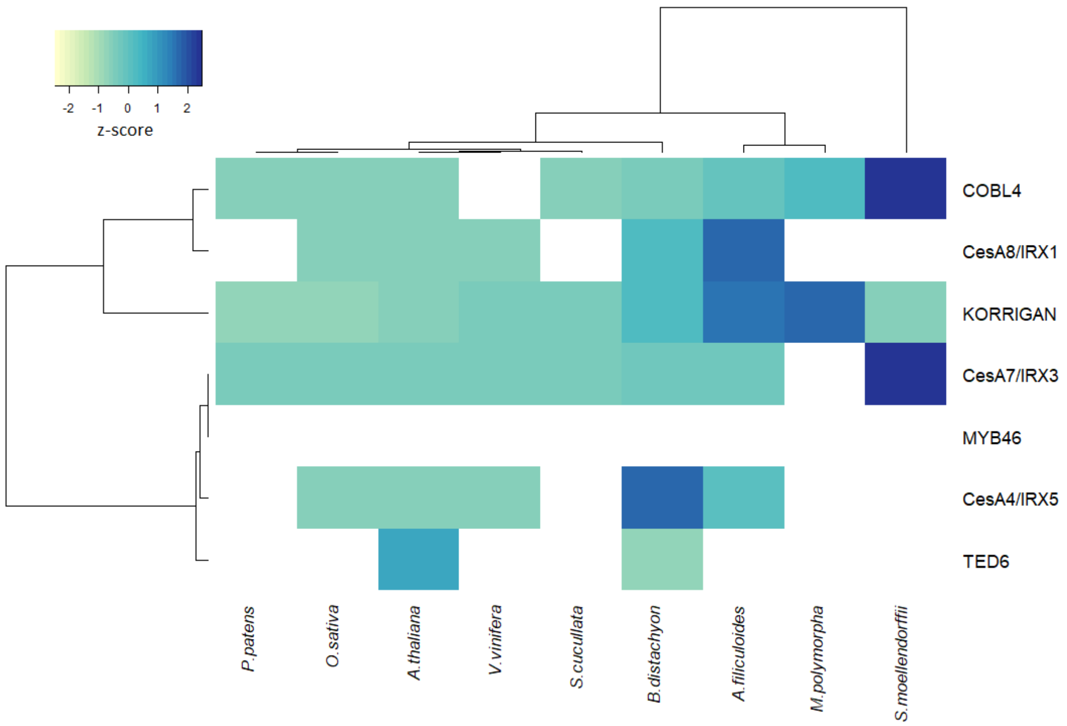

Supplemental Figure S: RNA-seq expression heat map (TPM) of orthologous xylan and cellulose biosynthesis genes involved in vasculature development. Average TPM expression values were derived from the same RNA-seq datasets used for analysing dicistronic tRNA-mRNA transcription. Colour represents the expression Z-score (TPM minus mean over s.d) of each gene (row) in a given species (column). A white panel is used to indicate cases where unequivocal ortholog assignment was not possible or wherean orthologous gene member could not be identified.
