# Supplementary material for: Tissue and regional expression patterns of dicistronic tRNA–mRNA transcripts in grapevine (Vitis vinifera) and their evolutionary co-appearance with vasculature in land plants
Source: Hortic Res. 2021 Jun 1;8:137. doi: 10.1038/s41438-021-00572-5 (PMC8166872; doi:10.1038/s41438-021-00572-5)
Supplement: Supplementary file 11 — Supplementary table S2 [file 41438_2021_572_MOESM11_ESM.docx]

| Supplementary table S2: Percentage of each tRNA isotype expressed in leaf and berry samples. | | |  |
| --- | --- | --- | --- |
|  |  |  |  |
| Isotype | Leaf % | Berry % |  |
| Pro | 16% | 18% |  |
| Ala | 9% | 9% |  |
| Arg | 8% | 8% |  |
| Gly | 8% | 6% |  |
| Gln | 6% | 4% |  |
| Asn | 5% | 4% |  |
| Thr | 5% | 7% |  |
| Met | 5% | 4% |  |
| Val | 5% | 6% |  |
| Leu | 5% | 2% |  |
| His | 4% | 3% |  |
| Ile | 4% | 4% |  |
| Cys | 3% | 4% |  |
| Phe | 3% | 3% |  |
| Tyr | 3% | 3% |  |
| Ser | 3% | 4% |  |
| Glu | 3% | 4% |  |
| Asp | 2% | 1% |  |
| Lys | 1% | 1% |  |
| Trp | 0% | 1% |  |
