# Supplementary material for: Tissue and regional expression patterns of dicistronic tRNA–mRNA transcripts in grapevine (Vitis vinifera) and their evolutionary co-appearance with vasculature in land plants
Source: Hortic Res. 2021 Jun 1;8:137. doi: 10.1038/s41438-021-00572-5 (PMC8166872; doi:10.1038/s41438-021-00572-5)
Supplement: Supplementary file 15 — Supplementary table S6 [file 41438_2021_572_MOESM15_ESM.pdf]

Supplemental Table S6: List of primers used for cDNA synthesis and RT-PCR

Name	primer	Sequence	Comments	Tissue expressed	Use for:		
					cDNA synthesis	RT-PCR	Sanger sequencing
tRNA^{ProTGG}_F	Forward	TGCGAGAGGTCCCGAGTTCGATT	PCR product is 172 bp	Leaf & berry			
Intergenic_tRNA^{ProTGG}-VIT_18s0001g09050_R	Reverse	CTGTTTCCTTGCCTGCCACC					
VIT_18s0001g09050_R	Reverse	TGCATCATTGGCAGGATCCA					
tRNA^{ValCAC}_F	Forward	CACTAGAGGTCCCCGGTTCGAA	PCR product is 376 bp	Leaf			
Intergenic_tRNA^{ValCAC}-VIT_15s0046g02860_R	Reverse	CCGCAAGACCCAGATGGGAA					
VIT_15s0046g02860_R	Reverse	CCACCCCCTTTGAAGCCACA					
VvEF1-a_F	Forward	GAACTGGGTGCTTGATAGGC	PCR product is 150 bp	Leaf & berry			
VvEF1-a_R	Reverse	AACCAAAATATCCGGAGTAAAAGA					
tRNA^{GlyCCC}_R	Reverse	ACTAGATGCGCTGGATGAGG		Berry			
VIT_19s0177g00220_F	Forward	TGGGACTTTAGTGTGGCAAA	PCR product is 367 bp				
Intergenic_tRNA$^{GlyCCC-}$VIT_19s0177g00220_R	Reverse	TTTATGGTCCCTTTCCATGC					
