# Supplementary material for: Tissue and regional expression patterns of dicistronic tRNA–mRNA transcripts in grapevine (Vitis vinifera) and their evolutionary co-appearance with vasculature in land plants
Source: Hortic Res. 2021 Jun 1;8:137. doi: 10.1038/s41438-021-00572-5 (PMC8166872; doi:10.1038/s41438-021-00572-5)
Supplement: Supplementary file 17 — Supplementary table S8 [file 41438_2021_572_MOESM17_ESM.pdf]

Supplementary Table S8: Lignin related genes

	A.thaliana	*V. vinifera*	*O. sativa*	*B.distachyon*	*A. filiculoides*	*S. cucullata*	*S.moellendorffii*	*P. patens*	*M. polymorpha*
**PAL1**	At2g37040	GSVIVT01024292001	LOC_Os02g41650	Bradi3g49280	Azfi_s0009.g011807	Sacu_v1.1_s0091.g018764	424403	Pp3c14_11870V3.1.p	Mapoly0070s0071/Mp4g14110
**PAL2**	At3g53260	?	LOC_Os04g43800	Bradi5g15830	Azfi_s0063.g035303	Sacu_v1.1_s0253.g026791	n.d.	Pp3c1_18940V3.1.p	Mapoly0070s0068/Mp4g14140
**PAL4**	At3g10340	GSVIVT01025214001	LOC_Os02g41680	Bradi3g49260	Azfi_s0096.g043679	Sacu_v1.1_s0017.g007129	404263	Pp3c1_18830V3.1.p	Mapoly0014s0211/Mp1g10150
**C4H**	At2g30490	GSVIVT01024554001	LOC_Os05g25640	Bradi2g53470	Azfi_s0333.g065437	Sacu_v1.1_s0039.g012203	175973	Pp3c4_21680V3.1.p	Mapoly0163s0018/Mp6g00020
**4CL1**	At1g51680	GSVIVT01029182001	LOC_Os02g08100	Bradi3g05750	Azfi_s0114.g046013	Sacu_v1.1_s0149.g023264	171251	Pp3c18_6360V3.1.p	Mapoly0197s0014/Mp5g01200
**4CL2**	At3g21240	GSVIVT01029183001	LOC_Os06g44620	Bradi3g52350	Azfi_s0013.g013344	n.d.	177393	Pp3c19_13170V3.1.p	n.d.
**HCT**	At5g48930	GSVIVT01016053001	LOC_Os04g42250	Bradi5g14720	Azfi_s0005.g009338	Sacu_v1.1_s0010.g004618	152997	Pp3c2_29140V3.1.p	Mapoly0003s0277/Mp7g12690
**C3H1**	At2g40890	GSVIVT01025800001	LOC_Os05g41440	Bradi2g21300	Azfi_s0355.g066730	Sacu_v1.1_s0001.g000031	271465	Pp3c22_19010V3.1.p	Mapoly0037s0087/Mp3g11100
**CSE**	At1g52760	GSVIVT01017214001	LOC_Os02g11720	Bradi1g24490	Azfi_s0045.g030088	Sacu_v1.1_s0674.g027619	113971	Pp3c19_14430V3.1.p	Mapoly0032s0047/Mp5g13540
**CCoAOMT1**	At4g34050	GSVIVT01022100001	LOC_Os06g06980	Bradi1g48370	Azfi_s0002.g001302	Sacu_v1.1_s0016.g006795	80209	Pp3c23_5530V3.1.p	Mapoly0035s0134/Mp6g03550
**CCoAOMT7**	At4g26220	GSVIVT01015245001	LOC_Os08g38910	Bradi4g33340	Azfi_s0167.g054536	?	271191	?	Mapoly0099s0056/Mp7g01830
**CCR1**	At1g15950	GSVIVT01034241001	LOC_Os08g34280	Bradi3g36887	Azfi_s3139.g114683	Sacu_v1.1_s0016.g006953	271114	Pp3c7_17190V3.1.p	?
**F5H1**	At4g36220	GSVIVT01024186001	LOC_Os10g36848	Bradi3g30590	n.d.	n.d.	n.d.	n.d.	n.d.
**COMT**	At5g54160	GSVIVT01008854001	LOC_Os08g06100	Bradi3g16530	Azfi_s0019.g015239	Sacu_v1.1_s0056.g014618	438615	Pp3c12_5860V3.1.p	Mapoly0337s0001/Mp2g07360
**CAD3**	At4g34230	GSVIVT01003150001	LOC_Os02g09490	Bradi3g06480	Azfi_s0001.g000474	Sacu_v1.1_s0209.g025876	444096	?	?
**CAD6**	At4g37970	GSVIVT01006303001	LOC_Os10g29470	Bradi3g17920	Azfi_s0021.g015839	Sacu_v1.1_s0027.g009722	230239	Pp3c1_39700V3.1.p	?
**LAC4**	At2g38080	GSVIVT01034003001	LOC_Os11g48060	Bradi1g74320	Azfi_s0374.g067219	Sacu_v1.1_s0058.g014969	165365	Pp3c15_19050V3.1.p	n.d.
**LAC11**	At5g03260	GSVIVT01025694001	LOC_Os01g44330	Bradi2g54680	Azfi_s1432.g103020	Sacu_v1.1_s0039.g012147	404075	Pp3c1_13930V3.1.p	Mapoly0049s0002/Mp3g20310
**LAC17**	At5g60020	GSVIVT01025046001	LOC_Os01g62480	Bradi1g24880	Azfi_s0402.g068306	Sacu_v1.1_s0031.g010585	95740	Pp3c6_3290V3.1.p	n.d.

n.d. not detected

? unequivocal ortholog assignment not possible
