# Supplementary material for: Tissue and regional expression patterns of dicistronic tRNA–mRNA transcripts in grapevine (Vitis vinifera) and their evolutionary co-appearance with vasculature in land plants
Source: Hortic Res. 2021 Jun 1;8:137. doi: 10.1038/s41438-021-00572-5 (PMC8166872; doi:10.1038/s41438-021-00572-5)
Supplement: Supplementary file 18 — Supplementary table S9 [file 41438_2021_572_MOESM18_ESM.pdf]

Supplemental Table S9: Xylan related genes

	A. thaliana	*V. vinifera*	*O. sativa*	*B. distachyon*	*A. filiculoides*	*S. cucullata*	*S.moellendorffii*	*P. patens*	*M. polymorpha*
IRX9	At2g37090	GSVIVT01025636001	LOC_Os03g17850	Bradi2g37970	Azfi_s0008.g011358	n.d.	n.d.	Pp3c19_5960V3.1.p	n.d.
I9H/IRX9L	At1g27600	GSVIVT01009245001	LOC_Os10g13810	Bradi3g23120	Azfi_s0384.g067471	Sacu_v1.1_s0042.g012788	78265	Pp3c13_8430V3.1.p	Mapoly0113s0008/Mp1g02600
I14H/IRX14L	At5g67230	GSVIVT01022064001	LOC_Os06g47340	Bradi1g33320	Azfi_s0004.g008338	Sacu_v1.1_s0055.g014339	12082	Pp3c15_16210V3.1.p	Mapoly0004s0253/Mp3g14180
IRX10/GUT2	At1g27440	GSVIVT01008661001	LOC_Os01g70200	Bradi2g59400	Azfi_s0113.g045995	Sacu_v1.1_s0002.g000912	n.d.	n.d.	n.d.
IRX10L/GUT1	At5g61840	GSVIVT01001097001	LOC_Os02g32110	Bradi5g08400	Azfi_s0198.g057414	Sacu_v1.1_s0085.g018365	442111	Pp3c2_6740V3.1.p	Mapoly0002s0257/Mp1g26200
IRX15	At3g50220	GSVIVT01004862001	LOC_Os06g47310	Bradi3g04460	n.d.	n.d.	n.d.	n.d.	n.d.
IRX15L	At5g67210	n.d.	LOC_Os04g55640	Bradi5g24040	n.d.	n.d.	n.d.	n.d.	n.d.
ESK1	At3g55990	GSVIVT01032800001	LOC_Os03g18140	Bradi1g65530	n.d.	n.d.	n.d.	n.d.	n.d.
FRA8	At2g28110	GSVIVT01031485001	LOC_Os03g01760	Bradi1g78027	Azfi_s0017.g014552	Sacu_v1.1_s0029.g010231	442723	Pp3c3_17750V3.1.p	Mapoly0050s0126/Mp3g13340
F8H	At5g22940	n.d.	n.d.	Bradi3g32610	Azfi_s0074.g037347	Sacu_v1.1_s0018.g007380	5415	Pp3c4_17130V3.1.p	n.d.
IRX8	At5g54690	GSVIVT01014592001	LOC_Os12g38930	Bradi4g03670	n.d.	n.d.	n.d.	n.d.	n.d.

n.d.	not detected
?	unequivocal ortholog assignment not possible
