# Supplementary material for: Tissue and regional expression patterns of dicistronic tRNA–mRNA transcripts in grapevine (Vitis vinifera) and their evolutionary co-appearance with vasculature in land plants
Source: Hortic Res. 2021 Jun 1;8:137. doi: 10.1038/s41438-021-00572-5 (PMC8166872; doi:10.1038/s41438-021-00572-5)
Supplement: Supplementary file 19 — Supplementary table S10 [file 41438_2021_572_MOESM19_ESM.pdf]

Supplementary Table S10: Secondary cell wall celluolose related genes

	A. thaliana	*V. vinifera*	*O. sativa*	*B. distachyon*	*A. filiculoides*	*S. cucullata*	*S.moellendorffii*	*P. patens*	*M. polymorpha*
MYB46	At5g12870	n.d.	n.d.	n.d.	n.d.	n.d.	n.d.	n.d.	n.d.
CesA4/IRX5	At5g44030	GSVIVT01028402001	LOC_Os10g32980.1	Bradi3g28350	Azfi_s0007.g010884	?	?	?	n.d.
CesA7/IRX3	At5g17420	GSVIVT01023643001	LOC_Os09g25490.1	Bradi4g30540	Azfi_s0059.g034631	Sacu_v1.1_s0085.g018303	163575	Pp3c9_11990V3.1.p	n.d.
CesA8/IRX1	At4g18780	GSVIVT01021248001	LOC_Os01g54620.1	Bradi2g49912	Azfi_s0230.g059145	?	?	?	n.d.
KORRIGAN	At5g49720	GSVIVT01023102001	LOC_Os03g52630.1	Bradi1g09460	Azfi_s0006.g009785	Sacu_v1.1_s0149.g023273	75214	Pp3c3_27980V3.1.p	Mapoly0061s0043/Mp1g24780
COBL4	At5g15630	GSVIVT01036565001	LOC_Os03g30250.1	Bradi1g59880	Azfi_s0018.g014792	Sacu_v1.1_s0125.g021681	271954	Pp3c12_4550V3.1.p	Mapoly0057s0007/Mp7g06600
TED6	At1g43790	n.d.	n.d.	Bradi4g45531	n.d.	n.d.	n.d.	n.d.	n.d.

n.d.	not detected
?	unequivocal ortholog assignment not possible
