# Supplementary material for: Tissue and regional expression patterns of dicistronic tRNA–mRNA transcripts in grapevine (Vitis vinifera) and their evolutionary co-appearance with vasculature in land plants
Source: Hortic Res. 2021 Jun 1;8:137. doi: 10.1038/s41438-021-00572-5 (PMC8166872; doi:10.1038/s41438-021-00572-5)
Supplement: Supplementary file 20 — Supplementary table S11 [file 41438_2021_572_MOESM20_ESM.pdf]

Supplementary table S11: Number of tRNA-mRNA identified for each tRNA isodecoders in independent RNA-seq datasets (NCBI-SRA) in vascular and non-vascular land plants

Amino acid	Anticodon	*Selaginella moellendorfii*	*Salvinia cucullata*	*Azolla Filiculoides*	*Vitis vinifera*	*Arabidopsis thaliana*	*Brachypodium distachyon*	*Oryza sativa*	*Marchantia polymorpha*	*Physcomitrella patens*
Ala	AGC	1						*4*		
	CGC							*2*		
	TGC	2			2	*1*				
Arg	ACG	3		1						
	CCG				1					
	TCG	1								
	TCT	4						*1*		
	UCU	1								
Asn	GTT	2			2	*2*				
Asp	GTC				1					
Cys	GCA	2								
Gln	CTG				2	*1*	*1*	*1*		
Glu	CTC	2				*1*				
	TTC	3								
Gly	CCC				1					
	GCC							*1*		
	TCC				4					
His	GTG	2			1			*1*		
Ile	AAT				2			*1*		
	GAT	3								
Leu	CAA	2					*2*			
	CAG		2							
	TAA	1								
	TAG							*4*		

Lys	CTT							*2*		
	TTT	1								
Met	CAT	1						*1*		
Phe	GAA	3			5			*1*		
Pro	AGG	2				*1*				
	CGG	2			1					
	TGG				13	*1*				
Ser	CGA				1					
	TGA					*1*				
Thr	AGT				2	*1*		*2*		
Trp	CCA			3						
Tyr	GTA					*2*		*6*		
	GUA	2								
Val	AAC	4			1					
	CAC							*2*		
	GAC	1								
	TAC	4								
