# Supplementary material for: Tissue and regional expression patterns of dicistronic tRNA–mRNA transcripts in grapevine (Vitis vinifera) and their evolutionary co-appearance with vasculature in land plants
Source: Hortic Res. 2021 Jun 1;8:137. doi: 10.1038/s41438-021-00572-5 (PMC8166872; doi:10.1038/s41438-021-00572-5)
Supplement: Supplementary file 20 [file 41438_2021_572_MOESM12_ESM.docx]

**Supplementary table S3**: Sanger sequencing result of the RT-PCR product from the intrergenic region of two dicistronic candidates (cDNA)			PCR product were align to the query sequence in Blast2sequence with defult settings								
Name	primer	Sequencing results	Query sequence: tRNA + intergenic region	Target ID	seq primer	Sequence length	Max Score	Total Score	Query Cover	E value	Per. Ident
tRNA^{ProTGG}	Forward	>1_ProC_F_B09<br>TTTCTTTGGCTGGGTTTTGGTTTTACTTCACC<br>ATAAACCTCAAAAAAGCCCTCTTATGCT<br>CTTCTGCAAATTTCATTTGTGTTATTGGTACT<br>GAAACTCCGAGGCGGTGGCAGGCAAGGA<br>AACAG	">tRNA-Pro-TGG-2-9_Intergenic_region<br>TGCGAGAGGTCCCGAGTTCGATT<br>CTCGGAATGCCCCAATCTTTTTATTTTCTTTCGCTGGGTTT<br>TGGTTTTACTTCACCATAA<br>ACCTCAAAAAAGCCCTCTTATGCTCTTCTGCAAATTTCAT<br>TTGTGTTATTGGTACTGAAA<br>CTCCGAGGCGGTGGCAGGCAAGGAAACA"	tRNA-Pro-TGG-2-9_Intergenic_region	Forward (F)	172	224	224	0.72	9E-64	99.2%
Intergenic_tRNA^{ProTGG}-Vv18s0001g09	Reverse	>2_ProC_R_B10<br>TGAATTTGCAGAGAGCATAAGAGGGCTTTTT<br>TGAGGTTTATGGTGAAGTAAAACCAAAAC<br>CCAGCGAAAGAAAATAAAAAGATTGGGGCA<br>TTCCGAGAATCGAACTCGGGACCTCTCGCA	CGGGCATTTGGTCTAGTGGTATGATTCTCGCTTTGGGTGC<br>GAGAGGTCCCGAGTTCGATT<br>CTCGGAATGCCCCAATCTTTTTATTTTCTTTCGCTGGGTTT<br>TGGTTTTACTTCACCATAAACCTCAAAAAAGC<br>CCTCTTATGCTCTTCTGCAAATTTCATTTGTGTTATTGGTA<br>CTGAAACTCCGAGGCGGTG<br>GCAGGCAAGGAAA		Reverse (R)		213	213	0.69	6E-60	99.2%
tRNA^{ValCAC}	Forward	>5_ValC_F_C01<br>ATTGCCAC-<br>AGTCTTCCATTTCTGTTGGGAGTCTCCCAGGG<br>TCAGAGTATCAACGACACT<br>CAGTGCCACAATCTCATTTCCATTTCTGCTAG<br>GAGTCTCCCAGATTCTCAATATCAACAA<br>CACTCACCCAGATTTTTAACATTTTCTCATCT<br>GGATGTTCATCAATTAGTCAAACAATGC<br>AGATTCAGCCACACCCACTTCCAAACTATAGT<br>CTTAGGTCACCAATTTTCTCACCTTCGA<br>CCCATTTTCTCATACCTTTCTCATCATCATTCC<br>AACCTACTAAACTTGTCTCTATTCCAA<br>ATTTCCCATCTGGGTCTTGCG	">tRNA-Val-CAC-1-7_Intergenic_region<br>CACTAGAGGTCCCCGGTTCGAACCCGGGCTCAGACA<br>TTTGCATTTTTATTTTATTATTGCCAGAGTCTTCCATTTCT<br>GTTG<br>GGAGTCTCCCAGGGTCAGAGTATCAACGACACTCAGTGC<br>CACAATCTCATTTCCATTTCT<br>GCTAGGAGTCTCCCAGATTCTCAATATCAACAACACTCAC<br>CCAGATTTTTAACATTTTCT<br>CATCTGGATGTTCATCAATTAGTCAAACAATGCAGATTCA<br>GCCACACCCACTTCCAAACT<br>ATAGTCTTAGGTCACCAATTTTCTCACCTTCGACCCATTTT<br>CTCATACCTTTCTCATCAT<br>CATTCCAACCTACTAAACTTGTCTCTATTCCAAATTTCCCA<br>TCTGGGTCTTGCGG"		Forward (F)	376	586	586	0.85	6E-172	99.7%
Intergenic_tRNA^{ValCAC}-Vv15s0046g02860	Reverse	>6_ValC_R_C02<br>ATGAGAAAATGGGTCGAAGGTGAGAAAATT<br>GGTGACCTAAGACTATAGTTTGGAAGTGGG<br>TGTGGCTGAATCTGCATTGTTTGACTAATTGA<br>TGAACATCCAGATGAGAAAATGTTAAAA<br>ATCTGGGTGAGTGTTGTTGATATTGAGAATC<br>TGGGAGACTCCTAGCAGAAATGGAAATGA<br>GATTGTGGCACTGAGTGTCGTTGATACTCTG<br>ACCCTGGGAGACTCCCAACAGAAATGGAA<br>GACTGTGGCAATAATAAAATAAAAATGCAAA<br>TGTCTGAGCCCGGGTTCGAACCGGGGACC<br>TCTAGTG		tRNA-Val-CAC-1-7_Intergenic_region	Reverse (R)		562	562	0.81	1E-164	99.7%
